# Tourette Syndrome: A Mini-Review

**DOI:** 10.3389/fneur.2018.00139

**Published:** 2018-03-09

**Authors:** Michal Novotny, Martin Valis, Blanka Klimova

**Affiliations:** ^1^Biomedical Research Centrum, University Hospital Hradec Králové, Hradec Králové, Czechia; ^2^Department of Neurology, University Hospital Hradec Králové, Hradec Králové, Czechia; ^3^Faculty of Informatics and Management, University of Hradec Králové, Hradec Králové, Czechia

**Keywords:** Tourette syndrome, tic disorders, movement disorders, pharmacotherapy, quality of life

## Abstract

The purpose of this mini-review is to provide the latest information on epidemiology, pathophysiology, diagnosis, and treatment of Tourette syndrome (TS). The authors conducted a literature search of available sources describing the issue of tic disorders with special focus on TS and made a comparison and evaluation of relevant findings. The results of this mini-review indicate that TS is a complex disorder, which has a significant impact on the quality of life of both the patients and his/her family. Therefore, early and proper diagnosis and treatment are necessary in order to reduce or even eliminate both symptoms and social burden of the patient. This requires a multidisciplinary management approach in order to meet the patients’ special needs. Future research should focus on neuroimaging, new neurotransmitter targets, in functional neurosurgery, as well as the effect of non-pharmacological psychotherapies for these people.

## Introduction

Motor tics are among the most common dyskinetic manifestations. Their occurrence is mainly associated with primary tic disorders, including transient tic disorders, chronic tic disorders (motor or vocal), and Tourette syndrome (TS). TS is a neurodevelopment disorder described in detail in 1885 by the French neurologist Georges Albert Gilles de la Tourette, followed by a name (1).

Characteristic signs of the disorder are sudden repetitive motor and phonic tics. Clinically, this is a complex disorder in which the severity, frequency, fluctuation, and localization of speech and tics are highly individual (1) In clinical practice, the characteristic features of tics accompanying TS were found. Tics are thought to be unintentional, but they can be suppressed temporarily by will. However, there is a “rebound” phenomenon, when after suppression the accumulated tics break out at a greater intensity than is usual for the patient. The deterioration of tics (both in quality and quantity) can occur under stress, excitement, or fatigue. On the contrary, tic expressions recede when the patient is engaged in a mental or physical activity requiring concentration. Often there is an inner tension before the tics breaks out, and after performance of the tics, the patient feels relieved. Interestingly, tics can persist in all stages of sleep (1, 2). In addition, the disorder is often accompanied by psychiatric behavioral disorders, such as attention deficit hyperactivity disorder (ADHD), obsessive-compulsive disorder (OCD), self-injurious behavior, depression, or specific learning disabilities.

The aim of this mini-review is to provide the latest information on epidemiology, pathophysiology, diagnosis, and treatment of TS. This mini-review could also help destigmatize patients, raise awareness of disorder, and improve the quality of life of both the patients and their families.

## Methods

The authors conducted a literature search of available sources describing the issue of tic disorders with special focus on TS. Research studies were selected on the basis of research topics (such as tic disorders, movement disorders, TS and diagnosis, TS and treatment) found in the world’s acknowledged databases, such as Web of Science, PubMed, Springer, and Scopus from the period of 2010 up to the present time. These research studies were classified according to their relevancy. Most of these articles focused on movement disorders and tic disorders, fewer than on TS. The information found in the selected studies on TS, its epidemiology, pathophysiology, symptoms, diagnosis, and treatment was carefully evaluated and it is described and discussed in the following sections.

## Epidemiology

The symptoms of TS begin already in childhood and the estimated prevalence is 3–9/1,000 children younger than the school age. The average age at which the first episode of motor tics occurs is 4–6 years. TS is more common in boys than in girls with a prevalence of 3–4:1. The phonic tics predominantly follow the motor ones. In practice, primitive simplex sounds like sniffing and coughing are often mistakenly attributed to “allergies” at first. Literature states that the maximum in the severity of TS manifestations occurs at the age of 10–12 years. Most patients will have complete or nearly complete remission of the disorder after 21 years of age. In 10–20% of cases, TS symptoms fluctuate, persist, or even worsen (3–5). Occurrence of tics is often preceded by behavioral disorders–most commonly ADHD—at the age of 3 years. On the other hand, the first symptoms of OCD appear up to several years after the beginning of tics with the maximal severity in late adolescence. Both of these psychiatric comorbidities often persist until adulthood, even during TS remission (1, 4).

## Etiology and Pathophysiology

The cause of TS is not yet known. It is assumed that the development of the disorder is conditioned by the participation of the genetic and nongenetic (epidemiological) factors. TS belongs to polygenic hereditary disorders, where several different genes are involved in the disorder. Even though chromosomal aberrations rarely occurred in patients with TS, a clear genetic cause has not yet been revealed. The negative effects of the environment are mainly prenatal intakes (maternal stress in pregnancy, smoking, infections, fetal hypoxia) and stressful events in the child’s life (3, 5). In professional circles, the theory of autoimmune-mediated TS is also discussed (6).

Tourette syndrome pathophysiological mechanisms have not yet been elucidated. Many findings are indicative of organic origin. Although tics are at least partially influenced by will, neurophysiological studies show that tics are not driven by normal motor ways designed to control free movements. Tics are not preceded by common preparatory potential (Bereitschaftspotential) (7), and polysomnography has shown tics at various stages of sleep (8). Strong evidence shows changes in central neurotransmitters, especially in dopaminergic modulation (3, 5). Dopamine antagonists and depleters improve tics, while drugs that augment central dopaminergic activity worsen them. The most interesting hypothesis assumes that the underlying TS is a developmental disorder leading to dopaminergic *hyperinnervation* of the striatum. Anatomical and functional links between the basal ganglia and the limbic system can explain the current occurrence of tics and complex behavioral problems in TS (9, 10). It was proved that basal ganglia, particularly the caudatus nucleus and ventral striatolimbic complex played a significant role in the pathogenesis of OCD and primitive reproductive behavior (11). The influence of sex hormones on the development of these structures can explain the difference in TS among sexes, the exacerbation of puberty and estrogenic stages of the menstrual cycle, and the characteristic occurrence of complex motor and sound tics, as well as behavioral manifestations with sexual content (12).

## Diagnosis

Tic disorders can generally be diagnosed based on a careful history, family history, and targeted neurological examination. As it has been stated above, characteristic signs of the disorder are sudden repetitive motor and phonic tics. They can be classified as simple or complex and their divisions, including examples are summarized in Table 1 (3). For setting a careful diagnosis, Yale Global Tic Severity Scale (YGTSS) can be used to find out in detail what types of tics the patient experienced or were experiencing or at what age they were. Furthermore, the questionnaire evaluates tics in terms of severity as to number, frequency, intensity, complexity, interference, and also impairment. The result is either Total Tic Severity Score (0–50) or Total YGTSS Score (0–100).

**Table 1:** Division of tics.

Classification	Division	Description	Examples
Simple	Motor	Tic affects a single muscle or a group of muscles	Eye movements; nose, mouth, tongue movements, or facial grimacing; head jerks/movements; shoulder jerks/movements; arm or hand movements; leg, foot, or toe movements; abdominal/trunk/pelvis movements
	
	Phonic	Simple, incomplete sounds	Coughing, throat clearing, sniffing, whistling, animal or bird noises
Complex	Motor	Tic consists of a coordinated and progressive movement, which may in some cases be socially inappropriate	Touching, tapping, picking, evening-up, reckless behaviors, rude/obscene gestures, obscene finger/hand gestures, unusual postures, bending, or gyrating, such as bending over, rotating, or spinning on one foot, copying the action of another (echopraxia), tic-like behaviors that could injure, self-injurious tic-like behavior(s)
	
	Phonic	The patient pronounces words or sentences that make sense	Rude or obscene words or phrases (coprolalia), repeating what someone else said, either sounds, single words, or sentences. Perhaps repeating what’s said on TV (echolalia), repeating something the patient said over and over again (palilalia)

An important diagnostic indicator may be the non-localized, little specific and poorly described urge to move, the so-called “premonitory urge.” Patients most often perceive tics as irresistible, but consciously done. The intentional component of these movements can be a useful feature for differentiating tics from other repetitive behaviors (myoclonus, functional jerks, stereotypies). Perceptions and feelings preceding the motor tics are purely involuntary. This phenomenon is closely related to the possibility of cognitive behavioral intervention of tics (focused on exercising attention and coping with responses to compulsion). Two scaling methods have been developed to assess the premonitory urges: the Premonitory Urges for Tics Scale and the University of São Paulo Sensory Phenomena Scale (13).

The diagnostic TS criteria were specified in the 2012 American Pychiatrics Association Diagnostic and Statistical Manual of Mental Disorders (DSM-V). The diagnosis of TS can be stated if a patient has experienced multiple motor and one or more tics in a certain period, and they do not have to be present at the same time. The first exposure lasts for more than 1 year and the onset of the disorder is dated before the patient has reached the age of 18. At the same time, involuntary movements and sounds of other etiologies must be avoided. Conversely, the presence of other comorbidity (ADHD, OCD) is not required for the diagnosis of TS (1–3).

## Treatment

The detailed description of the TS has been going on for many decades and, as well as progress in the pathophysiology of the disorder, progress has been made in treatment. Initially, TS was considered a purely psychiatric illness, and, therefore, only psychotherapeutic procedures, and in particular psychodynamic, were used in its treatment. In the early 1960s of the past century, haloperidol was introduced into the therapy of tics as a representative of classical antipsychotics, and thus began the era of pharmacotherapy of the manifestations of the disorder. As the knowledge about the nature of the disorder was made more specific, behavioral therapy (BT) and deep brain stimulation (DBS) became important (2).

The first-line therapy should be socio-education on the benignity of tics, this education should be given to the patient, but also his/her family, teachers, and fellow-students. In the second line, doctors should go to rational pharmacotherapy that can run concurrently with neuropsychological interventions, but can be conducted without them. The last treatment option is neurosurgical treatment, especially DBS. This option should be reserved for patients who do not respond to neurocognitive interventions or pharmacotherapy, or those patients who respond to treatment, but have very serious side effects (2, 3, 14).

### Neuropsychological Interventions

The neuropsychological interventions may help increase self-esteem, relieves depressive feelings as a result of a disorder. The evidence based and the most common treatments seem to be habit reversal training (HRT), exposure and response prevention (ER), and comprehensive behavioral intervention (CBIT) (15–17). However, these therapies are not possible for cognitively impaired patients.

Habit reversal training consists of five key techniques: awareness training, development of a competing response, contingency management, relaxation training, and generalization of skills (18). ER is a method of cognitive BT and form of exposure therapy in which individuals confront their fears and discontinue their escape response (19). CBIT is a common structured therapy, which trains patients to become aware of their tics and teaches them specific behavioral strategies that reduce tics (20). In addition, patients are advised to do psychoeducation, which helps understand the nature of TS (21).

### Pharmacology

According to ESSTS, there are four reasons why a doctor should think that it is time for the second line of treatment—pharmacotherapy: (a) tics cause subjective discomfort to the patient (e.g., pain or injury), (b) tics cause permanent social problems (e.g., social isolation or bullying), (c) tics cause emotional problems (e.g., depression) in the patient, (d) tics cause functional interference (e.g., impairment of academic achievements) (2, 14).

The basic group of drugs for TS therapy is antipsychotics (especially dopamine receptor antagonists). However, some physicians are reluctant to prescribe them because they fear their extrapyramidal side effects, especially tardive dyskinesia. The most effective drugs seem to be antipsychotics haloperidol, pimozide, and risperidone. In some countries, tiapride and sulpiride are widely used. In recent years, the dualistic neuroleptic aripiprazole has come to the fore. It is characterized by a highly effective treatment of tic disorders with a much lower incidence of sedation and metabolic side effects compared to classical antipsychotics. As the first results show, a promising candidate for TS treatment could be cannabis or affect the cannabinoid system (2, 9, 22).

As the various neurotransmitter hypotheses gradually emerged, already introduced drugs were gradually studied, sometimes with greater, sometimes with smaller success. None of the drugs listed below, however, has been introduced into routine clinical practice. In the theory of GABAergic and cholinergic transmission, clonazepam and baclofen appeared to be the most potent. The effect of glutamate as an excitatory neurotransmitter was included in the studies with riluzole (inhibitor), d-serine (stimulant), *N*-acetylcysteine, and acamprosate. The most recently studied neurotransmitter system is the histamine system, which has been influenced by the pitolisant in the studies (2, 14).

### Surgery

In the past 10 years, doctors have been interested in functional neurosurgery in connection with TS treatment. Multiple targets have been investigated for DBS, GPi DBS as a surgical approach for improving medication-refractory TS: thalamus, globus pallidus—internal segment, anterior limb internal capsule/nucleus accumbens, and multiple targets. Growing experience with DBS and expanding evidence of its effectiveness and tolerability leads to the question which target is the best and which patients are suitable for this irreversible procedure. In 2015, the International DBS Database and Registry Study Group endorsed by the TS Association of USA have published an update on the treatment of TS with DBS. The recommendation states that the suitability of a patient for the DBS must be confirmed by a clinical practitioner dealing with TS in accordance with the DSM-V protocol. The question remains whether to include or exclude patients with associated psychiatric or neurological disorders. Tics disorders must be assessed as very serious causing disability; drugs are refractory to conservative treatment; expected patient compliance after treatment; psychological assessment of the patient whether he will tolerate surgical intervention, postoperative monitoring, and whether he understands that there may be a possibility of DBT failure. In addition to its own failure, the patient should be advised of possible complications associated with neurosurgery (bleeding and infections at the site of the electrode application). Even its own stimulation effect is not without complications. Some patients may experience sedation, abulia, fatigue, apathy, sexual dysfunction, and visual disturbances (2, 23).

## Discussion

As findings indicate, TS is a complex disorder, which has a significant impact on the quality of life of both the patients and his/her family. Nevertheless, although the disorder is generally lifelong and chronic, it is not a degenerative disorder, and patients can lead a normal life expectancy. Furthermore, TS does not impair intelligence (24).

The findings also reveal that the early and proper diagnosis and treatment are necessary in order to reduce or even eliminate both symptoms and social burden of the patient. The first-line treatment includes behavioral treatments, especially HRT, ER, and CBIT, which prove to be effective in the reduction of the number of tic expressions, tic severity, and level of distress associated with tic and improved these patient’s self-perception of his/her competences and skills of conducting everyday activities [cf. (15, 25)]. Nevertheless, as Whittington et al. (17) state, there is evidence that these treatments are effective, but there is no evidence for the combination of medication and these behavioral. In addition, there is currently no evidence that these interventions are sufficiently effective and safe to be considered as treatments. Ganos et al. (21) also report that behavioral treatments are aimed mainly at older patients/children and that they are not available to all centers due to a lack of well-trained therapists. In fact, TS due to its complexity of symptoms and comorbidity requires a multidisciplinary management approach in order to meet the patients’ special needs.

Black (9) reports that research on TS is beginning to grow from case series and small pilot studies into finding new avenues through large-scale collaborative projects. Nevertheless, these projects should solve the following TS issues highlighted by Hollis et al. (26): accessing specialist care and behavioral interventions, delay in diagnosis, importance of anxiety and emotional symptoms, lack of provision of information to schools, and inadequate information regarding medication and adverse effects. They also emphasize that future research on TS should focus on the use of modern information technologies which could make the diagnosis and access to treatment easier. One of such examples, which has been already implemented is TicHelper.com (“TicHelper”), an interactive online treatment program for youth with chronic tic disorders or TS and their parents. This treatment program is easily navigable, provides clear instructions and appropriate content, and thus it is a potentially useful dissemination tool to make CBIT more accessible to families and patients (27).

## Conclusion

Even though more than 130 years have passed, since the TS in 1885, there are still many unanswered questions. The exact cause of changes in brain tissue remains unknown. Similarly, highly effective, targeted, and safe therapy is still unavailable. Concerning TS therapy, there are still several controversial topics, their main denominator is the lack of clinically relevant studies or comparative studies. For these reasons, it is not entirely clear whether psychotherapy or pharmacotherapy should be the first line of treatment or whether cannabinoids in TS treatment should or should not be used (28).

Although TS is a relatively rare neurological movement disorder, it has an irreversible impact on patient’s and his/her psychosocial life in terms of quality. Since the communication is the key aspect of person’s everyday life, more education of the public should be done in this area with respect to the Gilles de la TS. Future research should focus on neuroimaging, new neurotransmitter targets, in functional neurosurgery, as well as the effect of non-pharmacological psychotherapies for these people.

## Author Contributions

MN, BK, and MV equally contributed to the drafting, analyses, and final version of the whole manuscript. All authors read and approved the final manuscript.

## Conflict of Interest Statement

The authors declare that the research was conducted in the absence of any commercial or financial relationships that could be construed as a potential conflict of interest.
